# Supplementary material for: Polymorphisms in Pfkelch13 domains before and after the introduction of artemisinin-based combination therapy in Southwest Nigeria
Source: PLoS One. 2025 Mar 31;20(3):e0316479. doi: 10.1371/journal.pone.0316479 (PMC11957316; doi:10.1371/journal.pone.0316479)

Samples: 12921
Bases: 1093
Average spacing: 12

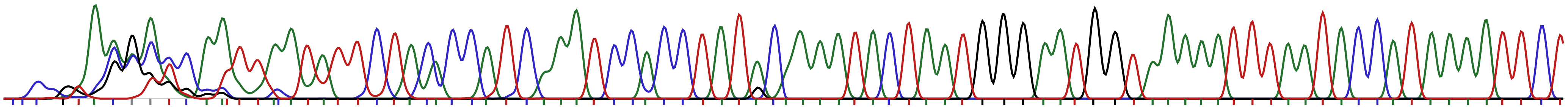

10 20 30 40 50 60 70 80 90

CC C G T A C - - T C A A T T T A C A T A T T C T A C A C C A T C A A A T C C A C C T A T A C A A A A T A C T A A T G G G A A T G G T A A A A T T T A A T A C C A T A A A A T T C T

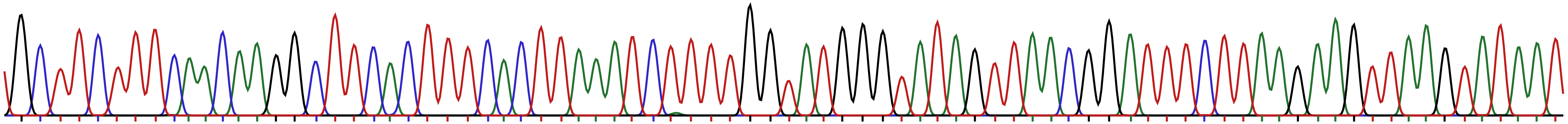

100 110 120 130 140 150 160 170

G C T T C T T T C A A C A A G G C T T C A C T T T C A C T T A A A T C T T T T G G T A T G G G T A T A G T T A A C G G A T T T C T T A A G A A G T T A A G T A T A A T

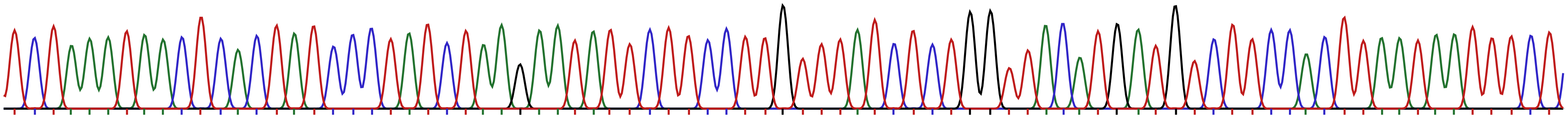

180 190 200 210 220 230 240 250

T C T A A A T A A C T C A C T A T C C C T A T C T A A G A A T A T T C T T C C T T G T T T A T C T C T G G T T A C A T G A T G T C T T C C A C T T A A T A A T T T C T

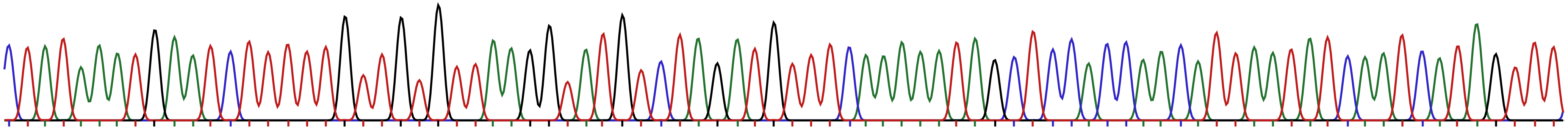

260 270 280 290 300 310 320 330 340

C T A T A A A T G A A T C T T T T T G T T G T G T T A A G G T A T G T C T A G A T G T T T C A A A A A T A G C T C C A C C A A C A T T A A T A T C A A T C A T A G T T T

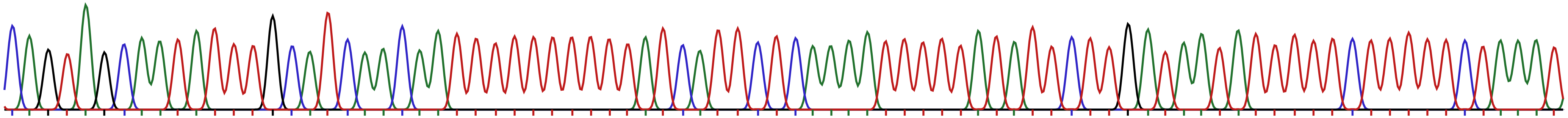

350 360 370 380 390 400 410 420

C A G T A G C A A T A T T T G C A T C A A C A A T T T T T T T T T T A T C A T T C T C A A A A T T T T T A T A T T C T T G A T A A T A T T T T T C T T T T T C T A A A T

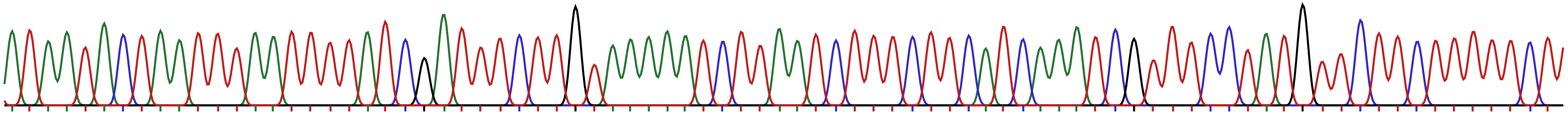

430 440 450 460 470 480 490 500

A T A A T A C T A A T T T A A T T T T A T C G A T T T C T T G T A A A A A T C T T A A T C T T T C T T C A T C A A A T C G T T T C C T A T G T T C T T C T T T T T C T

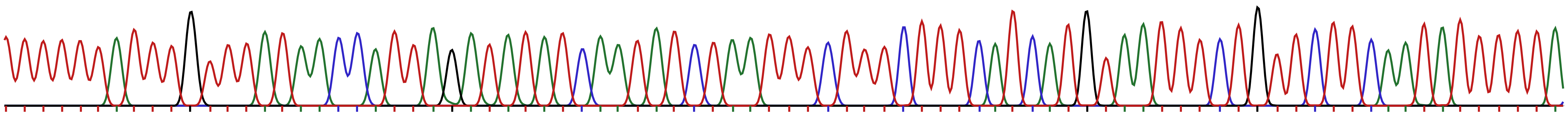

510 520 530 540 550 560 570 580 590

T T T T T T A T T T G T T T A T A A C C A T T A G A T A T A T C A A T A T C T A A T T T C T T T T C T T T T C A T C A T G T A A T T T C T G T T C T T C A A T A T T T T T A

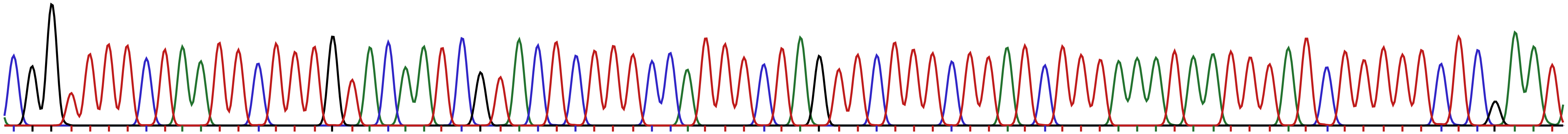

600 610 620 630 640 650 660 670

C G G T T T T C T A A T T C T T T G T A C A A T C G T A C T C T T T C C A T T T C T A G T T C T T T C T T A T C T T T A A A T A A T T T A T C T T T T T C T C G A A T

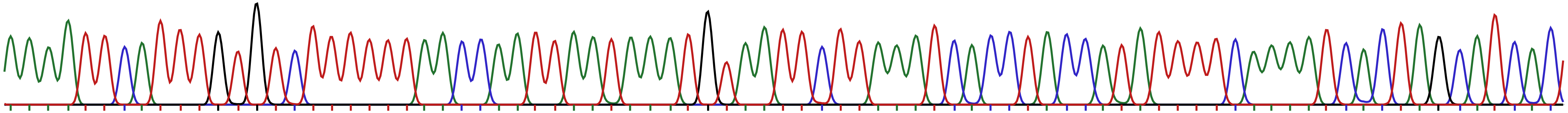

680 690 700 710 720 730 740 750

A A A A T T C A T T T G T G T C T T T T T T A A C C A A T T A A T A A A T G T A A T T C T T A A A T C A C C T A C C A T A T T T T C A A A A T C A C T A G C A T C A C

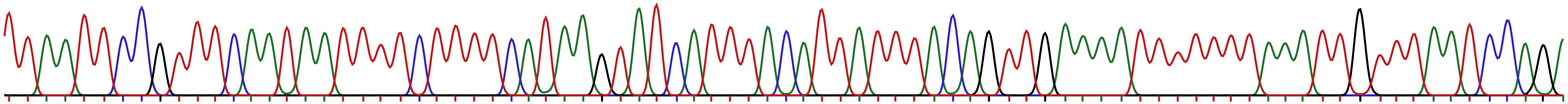

760 770 780 790 800 810 820 830 840

T T A A T T C C G T T T C A A T A A T T T T C T T T T C A T A A G T A T C A T T T A C A T T A T T T A C A G T T G A A A A T T T T T T T A A A T T G T T T A A T C C A G A

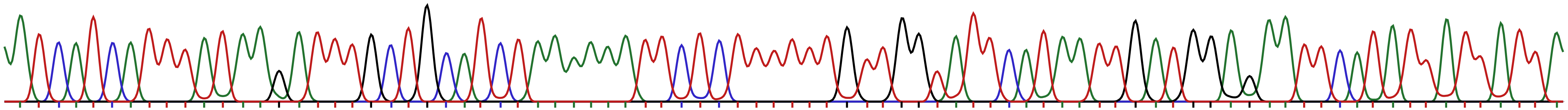

850 860 870 880 890 900 910 920

A T C A T C A T T T A T A A G A T T T G C T G C A T C T A A A A A A T T C T C T T T T T T G T T G G T A T T C A T A A T T G A T G G A G A A T T C A T A T T A T T A T T A

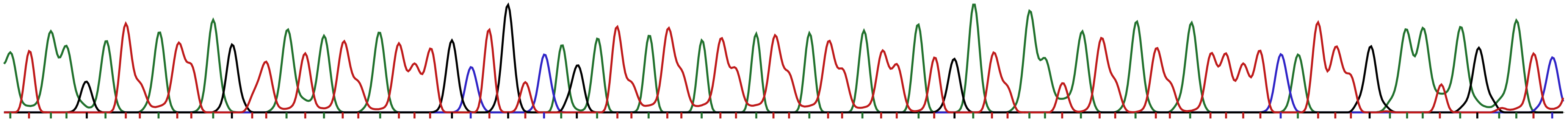

930 940 950 960 970 980 990 1000 1010

A T A A G A T T A T T A G T T A T A T T A T T T G C T G T C A G A T T A T T A T T A T T A T T A T T A T T A T T A T G A T T A A T A T T A T T A T T T T C A T T T G A A A T A G A A T C

Samples:	12921
Bases:	1093
Average spacing:	12

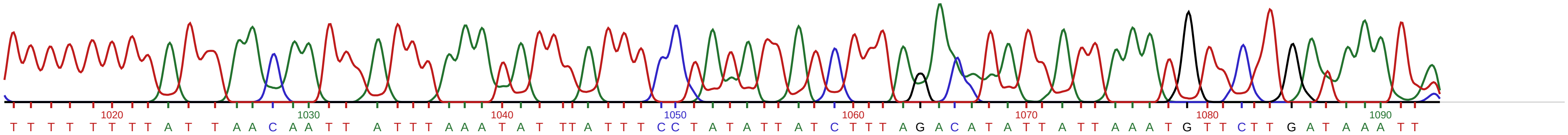

Supplement: Supporting information 2 — (ZIP) [file pone.0316479.s002.zip › 142KN2R_PREMIX_Plate_KELCH2_H03.pdf]
